# Sleep Disturbances Rate among Medical and Allied Health Professions Students in Iran: Implications from a Systematic Review and Meta-Analysis of the Literature

**DOI:** 10.3390/ijerph17031011

**Published:** 2020-02-05

**Authors:** Mojtaba Khaksarian, Masoud Behzadifar, Meysam Behzadifar, Firuzeh Jahanpanah, Ottavia Guglielmi, Sergio Garbarino, Paola Lanteri, Tania Simona Re, Riccardo Zerbetto, Juan José Maldonado Briegas, Matteo Riccò, Nicola Luigi Bragazzi

**Affiliations:** 1 Razi Herbal Medicines Research Center & Physiology Department, Lorestan University of Medical Sciences, Khorramabad 6814993165, Iran; mojkhaksar@yahoo.com; 2 Social Determinants of Health Research Center, Lorestan University of Medical Sciences, Khorramabad 6813833946, Iran; masoudbehzadifar@gmail.com (M.B.); firuzehjahanpanah@yahoo.com (F.J.); 3 Health Management and Economics Research Center, Iran University of Medical Sciences, Tehran 1449614535, Iran; m_behzadifar67@yahoo.com; 4 Department of Neuroscience, Rehabilitation, Ophthalmology, Genetics, Maternal and Child Health (DINOGMI), University of Genoa, 16132 Genoa, Italy; ottavia.guglielmi@gmail.com (O.G.); sgarbarino.neuro@gmail.com (S.G.); 5 Neurophysiopathology Centre, Department of Diagnostics and Applied Technology, Fondazione IRCCS, Istituto Neurologico “C. Besta”, 20133 Milan, Italy; paolanteri@yahoo.it; 6 UNESCO Chair “Health Anthropology Biosphere and Healing Systems”, University of Genoa, 16132 Genoa, Italy; tania.re77@gmail.com; 7 GESTALT Study Center (CSTG), 20129 Milano, Italy; r.zerbetto@cstg.it; 8 Department of Psychology and Sociology of Education, University of Extremadura, 06006 Badajoz, Spain; juanjose.maldonadob@gmail.com; 9 Azienda USL-IRCCS di Reggio Emilia, Dipartimento di Sanità Pubblica, Servizio di Prevenzione e Sicurezza degli Ambienti di lavoro (Department of Public Health, Occupational Health and Safety Services), 42122 Reggio Emilia, Italy; mricco2000@gmail.com; 10 Postgraduate School of Public Health, Department of Health Sciences (DISSAL), University of Genoa, 16132 Genoa, Italy; 11 Department of Mathematics and Statistics, Laboratory for Industrial and Applied Mathematics (LIAM), York University, Toronto, ON M3J 1P3, Canada

**Keywords:** sleep disturbances, sleep quality, Pittsburg Sleep Quality Index (PSQI), medical students, academic and clinical performance, Iran, systematic review and meta-analysis

## Abstract

Medicine and healthcare professions are prestigious and valued careers and, at the same time, demanding, challenging, and arduous jobs. Medical and allied health professions students, experiencing a stressful academic and clinical workload, may suffer from sleep disturbances. In Iran, several studies have been conducted to explore the prevalence rate among medical and healthcare professions students. The aim of this systematic review and meta-analysis was to quantitatively and rigorously summarize the existing scholarly literature, providing the decision- and policy-makers and educators with an updated, evidence-based synthesis. Only studies utilizing a reliable psychometric instrument, such as the Pittsburgh sleep quality index (PSQI), were included, in order to have comparable measurements and estimates. Seventeen investigations were retained in the present systematic review and meta-analysis, totaling a sample of 3586 students. Studies were conducted between 2008 and 2018 and reported an overall rate of sleep disturbances of 58% (95% confidence interval or CI 45–70). No evidence of publication bias could be found, but formal analyses on determinants of sleep disturbances could not be run due to the dearth of information that could be extracted from studies. Poor sleep is highly prevalent among Iranian medical and healthcare professions students. Based on the limitations of the present study, high-quality investigations are urgently needed to better capture the determinants of poor sleep quality among medical and healthcare professions students, given the importance and the implications of such a topic.

## 1. Introduction

Medicine and allied health professions represent prestigious and esteemed careers, but they are at the same time challenging, arduous, and demanding jobs, which require serious commitment, strenuous study, and high levels of motivation and dedication [1]. Medical and healthcare professions students experience a stressful academic and clinical workload, in terms of study hours, lessons, seminars, workshops, internships to attend, and examinations to take. Moreover, the environment is particularly competitive and aggressive ambition has become a highly pervasive phenomenon. Taken together, these factors contribute, at least partially, to explain the poor sleep quality experienced by the majority of medical and healthcare professions students, who tend to reduce the amount of hours they sleep in an attempt to adjust and cope with their workload, stringent deadlines, and the harsh environment in which they live. 

Unfortunately, this may have a dramatic impact on the current academic and clinical performance, on the learning process, and potentially on future medical activities [2], as well as on health outcomes in general. Poor sleep, indeed, impairs memory and alertness, and results, if chronic, in relevant psychological distress and diseases (including anxiety disorder and depression) and in a severe burden of cardiovascular co-morbidity [3,4,5,6,7]. 

Generally, a significant proportion of university students tend to be chronically sleep-deprived, sleeping less than 7 hours per night on week days, especially during the first year of university courses and in the weeks before an examination [3]. In a recently published global literature review specifically focusing on the medical students’ sleep experiences, authors found that poor sleep among medical students represents an emerging, serious public health concern. Indeed, poor sleep quality was not only a commonly reported issue among medical students, but its prevalence rate was also found to be higher than in the general population and in non-medical students [4]. 

Several variables, such as medical and allied health professions students’ attitudes, scarce awareness of one’s own condition of sleep deprivation and/or poor sleep hygiene, academic workload, and clinical demands have been identified as possible causative factors, profoundly affecting sleep patterns and schedules, but other potential mechanisms may also lead to sleep disturbances. In general, the precise etiology of sleep problems among medical and healthcare professions students is poorly understood [3,4,5,6,7]. Paradoxically, the amount of hours dedicated to sleep decreases as medical students progress from pre-clinical to clinical courses and their knowledge of sleep and sleep-related pathologies is expected to increase [6,7]. There seems to be a gap between medical and allied health professions students’ knowledge of sleep and the awareness of its importance, which reflects in poor sleep hygiene and unhealthy practices and lifestyles.

On the other hand, there is evidence that USA medical students do not necessarily possess high knowledge of sleep, and as such, their knowledge could be improved with sleep education that is not typically included in their medical curriculum [8,9]. Furthermore, medical and healthcare professions students may have a certain level of knowledge concerning the importance of sleep and may be aware that their sleep is disrupted, but they could choose not to prioritize their sleep-related health because of the demands of their medical training and/or personal lives. Additionally, everyday stressors that might be attributed to the demands of their medical training and/or personal lives may be impeding their ability to sleep even though they are knowledgeable and aware of the importance of sleep to them.

Currently, in the existing scholarly literature, few studies address the topic of poor sleep quality among medical and allied health professions students. As such, further studies and, in particular, epidemiological surveys are warranted to better understand the etiopathogenesis underlying sleep problems among medical and healthcare professions students and trainees, in order to effectively improve the overall quality of medical and allied health professions students’ lives, impacting as well on their academic and clinical performance. 

Sleep plays, indeed, an important role in the daily life of healthcare professions students, and is implied in the occupational risks of the future doctor and allied health professional (including night work, work-related stress and burn-out, workplace violence, among others) [10]. Furthermore, sleep is also relevant for the immunological and mental health of students [11,12,13].

In Iran, some studies have been conducted among medical and healthcare professions students, exploring their sleep experience and perceived quality. Therefore, the aim of the present systematic review and meta-analysis was to provide an updated, rigorous synthesis of the currently available scholarly literature on the topic. Meta-analytical studies are of crucial importance for various stakeholders, in that by pooling together primary studies we are able to overcome the limitations that plague the single studies included (in terms of small sample sizes and limited statistical power). As such, the present article has important, practical implications for educators as well as decision- and policy-makers, in that it could contribute to reshaping and re-scheduling of university timetables in order to meet with medical and allied health professions students’ sleep needs and requirements, developing and implementing ad hoc interventions and educating them to adopt healthier lifestyles. Furthermore, the present review is useful also for the scholarly community, offering a state-of-art snapshot of what is currently known and still unknown, and indicating future research directions and prospects. 

In this study we focused on the Iranian population, because Iran is one of the Asian countries for which several surveys are available, as noted by Azad and collaborators [4]. This work is the first of a series of investigations systematically appraising sleep disturbances among medical and healthcare professions students at the country level.

## 2. Materials and Methods 

This systematic review and meta-analysis adhered to the “Preferred Reporting Items for Systematic Reviews and Meta-Analyses” (PRISMA) guidelines, which contain an evidence-based minimum set of items for ensuring a proper reporting in systematic reviews and meta-analyses in a transparent, reproducible fashion [14]. 

The study protocol was designed a priori and devised using the “Preferred Reporting Items for Systematic review and Meta-Analysis—Protocols” (PRISMA—P) guidelines. It is available upon formal request to the Corresponding Author (N.L.B.).

The review title “Sleep disturbances among medical and allied health professions students in Iran: implications from a systematic review and meta-analysis of the literature” was guided by the so-called “PCC” mnemonic (namely, Population, Concept, and Context) suggested by the Joanna Briggs Institute (JBI). Structuring the title according to the PCC mnemonic enables us to clearly reflect and incorporate the core information about the focus and scope of the review.

The research review questions were generated after an extensive consultation of the research team. In more detail, the review questions were: (i) What is the prevalence rate of sleep disturbances among Iranian students attending medical schools and universities? (ii) What are the main determinants of the prevalence rate of sleep disturbances among Iranian medical and healthcare professions students? The study aim was to synthesize the existing scholarly literature concerning the prevalence rate of sleep disturbances among Iranian medical and allied health professions students and their determinants. Quantitative findings were presented by means of charts, tables, and figures, together with a detailed narrative report of the included studies.

Concerning the search strategy, several scholarly international databases, namely the ISI/Web of Science (WoS), PubMed/MEDLINE, Scopus, the “Directory of Open Access Journals” (DOAJ) and Embase, as well as Iranian bibliographic thesauri and repositories (namely, MagIran, the “Scientific Information Database” (SID) and Barakatkns), have been extensively searched from 1 January 2000 to 1 January 2020. 

The search strategy used a string that included relevant keywords, properly connected by Boolean operators, and was as follows: (Sleep disorders OR Sleep disturbances OR Sleep Pattern OR Sleep disruption OR Sleep Quality OR Insomnia OR Parasomnia disorders OR waking OR Pittsburgh Sleep Quality Index OR PSQI OR sleep deprivation) AND (Medical Students OR (Students AND Medical school)) AND Iran. 

The reference list of each eligible article was also reviewed to get related articles. Two authors (M.K. and Ma.B.) independently searched the databases. Google Scholar was also searched to strengthen the search strategy, in order: (i) to obtain the full text of articles, (ii) to retrieve further potentially relevant articles, and (iii) to screen the gray literature, in such a way to curb/minimize the risk of missing eligible studies. 

The main outcomes of this study were: (i) the prevalence rate of sleep disturbances among Iranian medical students, and (ii) their determinants. Inclusion and exclusion criteria were defined a priori before starting the literature search. In more detail, they were devised according to the Population/patients—Intervention/exposure—Comparison/comparator—Outcome/outcomes—Study design (PICOS) criteria. 

Inclusion criteria were the following: namely, (i) P (population/patients): studies investigating Iranian students; (ii) I (intervention/exposure): studies investigating students attending medical schools and universities; (iii) C (comparison/comparator): any type of comparison; (iv) O (outcome/outcomes): studies reporting the prevalence rate of sleep disturbances and their determinants or studies whose data allowed the calculation of the prevalence rate, using reliable, validated psychometric tools, such as the “Pittsburg Sleep Quality Index” (PSQI), in such a way as to have comparable measurements/estimates; (v) S (study design): studies whose design was observational, (vi) studies published either in Persian or in English; and, finally, (vii) studies published in peer-reviewed journals. 

Exclusion criteria were: (i) P: studies conducted among non-Iranian students; (ii) I: studies investigating students not attending medical schools and universities; (iii) O: studies whose data were not clear or not sufficiently detailed for the calculation of the prevalence rate or studies utilizing self-developed, not validated instruments/questionnaires.; (iv) S: studies that published case-reports or case-series or were designed as abstracts of communications presented at conferences; and, finally, (v) studies that investigated overlapping/duplicate populations. 

Concerning the data extraction process, two authors (M.K. and Ma.B.) independently extracted relevant information after finding and retrieving the studies. In cases of disagreement, a third author (N.L.B.) acted as the final referee to resolve the dispute. The surname of the first author of the study, the year of publication, the province of the study, the language of study, the number of participants, the tool used to report sleep disturbances, the prevalence reported in the study, and the number of people with sleep disturbances were extracted. An ad hoc designed Excel spreadsheet was utilized for properly reporting data extracted. The data extraction process was first pilot tested on a small pool of articles and, when the research team had achieved familiarization with the available literature and the research review questions, the process was extended and applied to the entire set of included articles.

For the quality assessment, the “STrengthening the Reporting of OBservational studies in Epidemiology” (STROBE) checklist was used to report and critically appraise the methodological aspects of included studies [15]. Two authors (M.K. and Ma.B.) independently evaluated the quality of each retained article. Each item of the STROBE checklist was thoroughly evaluated for each study and the total score obtained was ranked in three groups (namely, (i) 1 to 8, (ii) 9 to 16, and (iii) 17 to 22, reflecting weak, moderate, and good quality, respectively). Any discrepancies or different opinions were resolved by involving a third person (N.L.B.), acting as a judge. 

The research team involved in the current systematic review and meta-analysis was highly multi-disciplinary and combines different expertise, involving an expert neuroscientist (M.K.), three expert research methodologists (Ma.B., Me.B., and F.J.), one expert biostatistician and epidemiologist (N.L.B.), two sleep disorder experts (S.G. and O.G.), an expert neurophysiologist (P.L.), an expert occupational and public health physician (M.R.), an expert psychiatrist (R.Z.), an expert medical anthropologist (T.S.R.), and an expert psychologist (J.J.M.B.).

Concerning the statistical analysis, in order to calculate the prevalence rate of sleep disturbances among Iranian medical and allied health professions students, the DerSimonian–Laird random-effects model with its 95% confidence interval (CI) was applied [16]. The I^2^ test was used in order to quantitatively evaluate heterogeneity among studies [17]. Publication bias was assessed by both visually inspecting the symmetry of the funnel plot and computing the Egger’s linear regression test [18]. In order to quantitatively evaluate possible sources of heterogeneity among studies, meta-regression analyses were carried out, using sample size, year of publication, and average age of participants as independent predictors. Sub-group analyses were carried out based on study methodological quality, sample size, and study region. Furthermore, sensitivity analysis was used to ensure the stability and reliability of the results [19]. 

Figures with *p*-values less than 0.05 were considered to be statistically significant. The commercial software Stata version 12.0 (Stata Corp, College Station, TX, USA) was used to analyze all the collected and extracted data.

## 3. Results

The initial literature search yielded 142 studies (115 from the previously mentioned databases, bibliographic thesauri and repositories, and 27 from additional sources, including extensive cross-checking and cross-referencing and scanning of existing reviews on the topic). Duplicate studies were deleted after entering the list of retrieved items in the commercial EndNote Version 7.0 software (Thomson Reuters, Toronto, ON, Canada). A pool of 67 studies was then removed during this step. 

Subsequently, the authors reviewed the title of each eligible study and deleted 31 unrelated studies. At total of 44 studies were further screened in-depth and only 27 of them were selected and retained in the qualitative synthesis. A total of 10 studies were excluded because they were deemed irrelevant based on the research review questions (n = 6), did not report relevant data (n = 2) or prevalence data (n = 2). These studies did not use the PSQI, but rather self-report questionnaires. Finally, 17 investigations were included in the meta-analytical part of the study [20,21,22,23,24,25,26,27,28,29,30,31,32,33,34,35,36]. Two studies reported the mean scores of the PSQI indicating poor sleep quality but not enough quantitative details enabling computation of the prevalence rate of sleep disturbances, and were therefore excluded with reason from the quantitative synthesis. 

**Figure 1:**
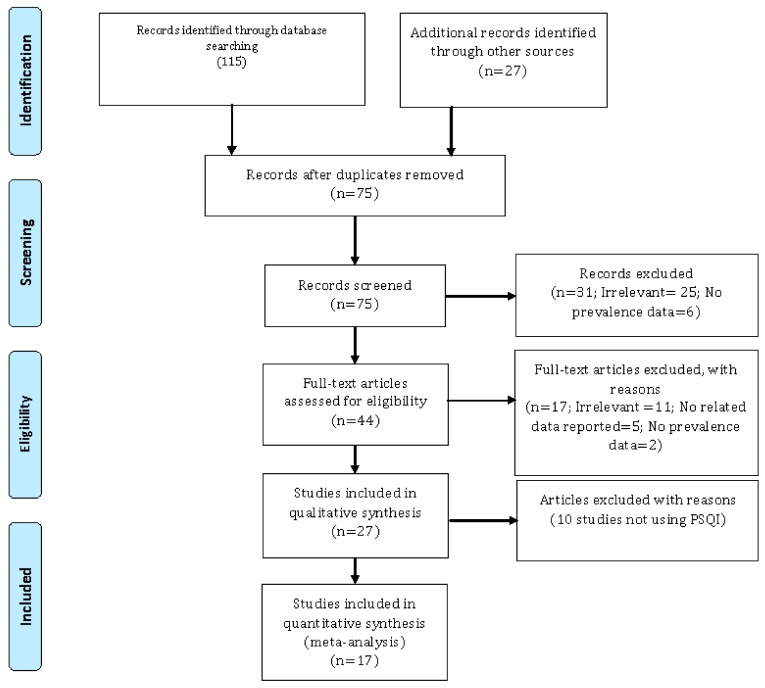
The process of study search, retrieval, and inclusion adopted in the present systematic review and meta-analysis concerning the prevalence rate of sleep disturbances among Iranian medical and allied healthcare professions students. Only studies utilizing the “Pittsburg Sleep Quality Index” (PSQI) were retained.

The study search and selection process is pictorially shown in Figure 1.

The characteristics of the studies selected are shown in Table 1. 

**Figure 2:**
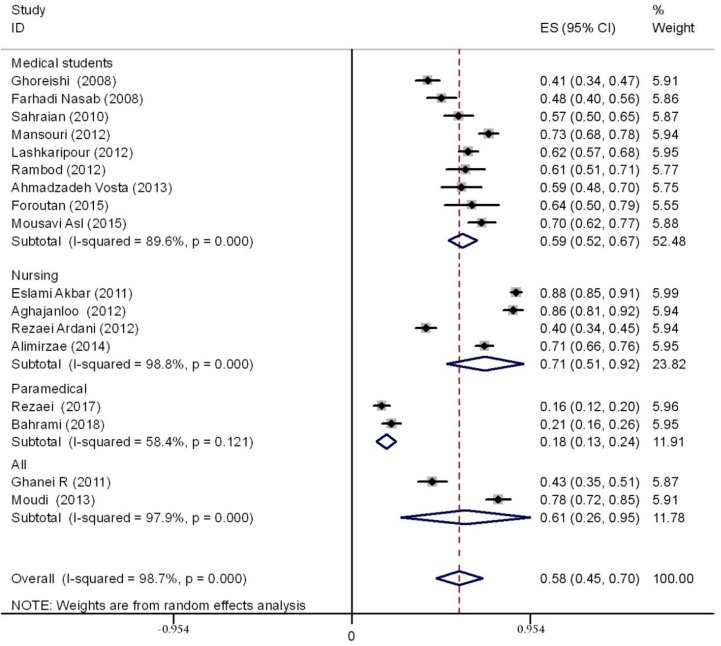
The forest plot showing the overall prevalence rate of sleep disturbances among Iranian medical and other allied health professions students and stratified by specific course degree. CI—confidence interval.

A total sample of 3586 students was evaluated. Studies were conducted between 2008 and 2018. Based on the random-effects model (high, statistically significant amount of heterogeneity, I^2^ = 98.7%), the prevalence rate of sleep disturbances was 58% (95%CI 45–70) in studies using the PSQI tool. Stratifying based on the specific degree course, the prevalence was 59% (95% CI 52–67) among medical students, 18% (95% CI 13–24) among paramedical students, 71% (95% CI 51–92) among nursing students, and 61% (95% CI 26–95) among allied health professions students (in these last studies, no specification was available concerning the specific degree course). The overall rate and the prevalence rate stratified according to the degree course are shown in Figure 2.

**Figure 3:**
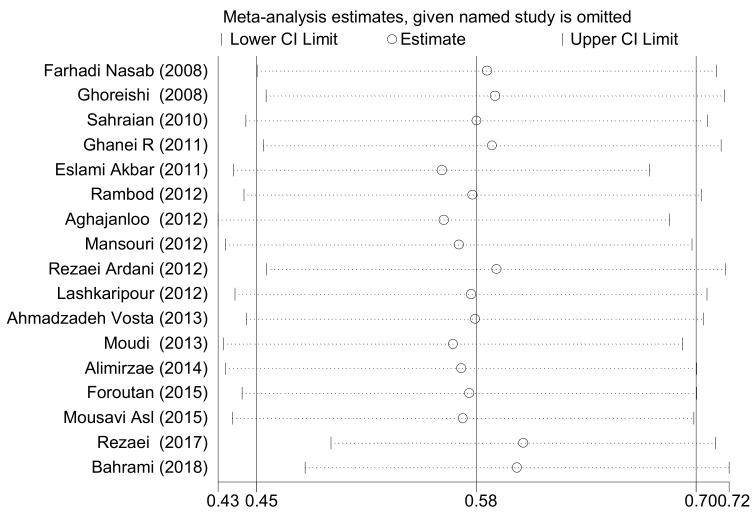
Sensitivity analysis showing the stability and reliability of the findings. The dotted line refers to the width of the 95% confidence interval.

In order to ensure the stability and reliability of our findings, sensitivity analysis was performed and the results before and after the analysis did not change, showing that the findings were stable. Figure 3 shows the sensitivity analysis. 

**Figure 4:**
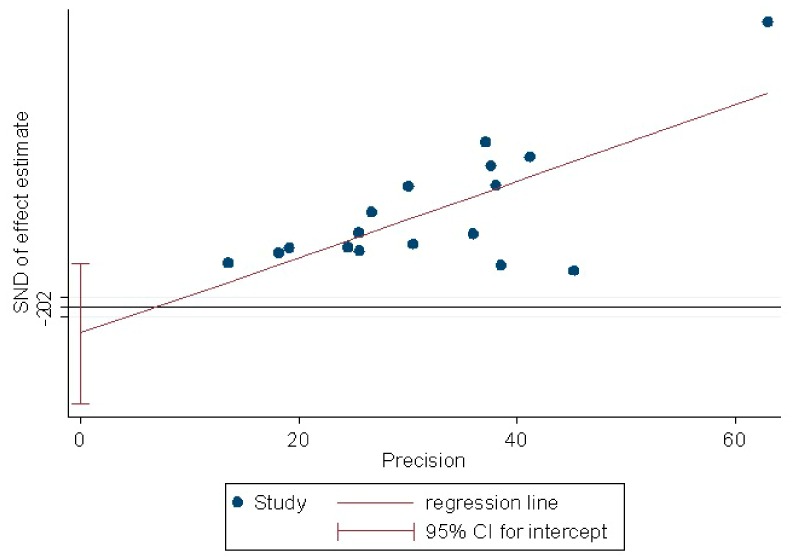
Publication bias as quantitatively assessed performing the Egger’s linear regression test. The finding of the test showed no evidence of publication bias. SND: standard normal deviate.

The Egger’s linear regression test yielded a *p*-value of 0.43, indicating that there was no evidence of publication bias (Figure 4). 

The results of meta-regression analyses based on sample size, year of publication, and average age of participants in the selected studies are presented in Table 2. The prevalence rate of sleep disturbances increased according to sample size and students’ average age, even though the effects were not statistically significant (*p* = 0.976 and *p* = 0.260, respectively). The prevalence rate of sleep disturbances decreased based on the year of study publication but, once again, this was not statistically significant (*p* = 0.308).

In conclusion, sleep disturbances were highly prevalent among Iranian medical and allied healthcare professions students. In particular, prevalence rates were high for medical and nursing students, whereas were lower among paramedical students. No impact of sample size, year of publication, and average age of students could be found. 

Concerning the quality appraisal, three studies were deemed of weak quality (weakness was noticed for several domains of the STROBE checklist, in particular reporting of introduction, methodology, and presentation of results), whereas five and nine investigations were judged of moderate and good quality, respectively. In order to better investigate sources of heterogeneity, sub-group analyses were performed. 

**Figure 5:**
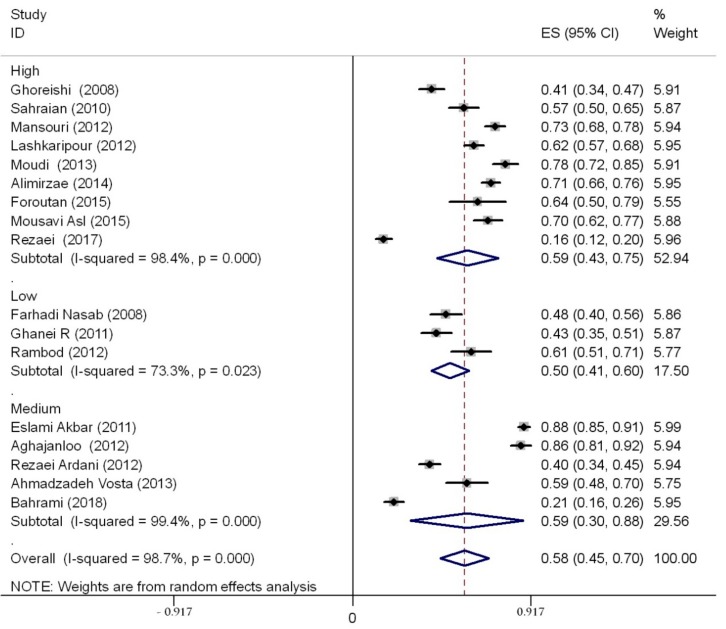
Sub-group analysis performed based on study quality.

The main findings are shown in Table 3—prevalence rates did not significantly vary based on methodological quality (Table 3 and Figure 5), sample sizes/number of participants recruited, and region in which the study was conducted. 

Even though a formal analysis of determinants of sleep disturbances among Iranian medical and allied health professions students was not possible because of the dearth of data, we attempted to extract the major factors impacting on sleep among the participants and we reported the number of studies per determinants and those reporting a significant association between the given determinant and sleep disturbances (Table 4).

## 4. Discussion

Sleep disturbances among students and, in particular, medical and healthcare professions students impair their academic performance, disrupt their social relationships, reduce their quality of life, and increase the risk of mental illnesses, such as depression and anxiety [36,37,38]. To the best of our knowledge, this is the first and comprehensive review of the prevalence rate of sleep disturbances among Iranian medical and allied health professions students. We found that poor sleep is a highly prevalent issue among them. 

To a certain degree, this finding is comparable with the existing literature on the topic—a recent meta-analytical study carried out in Brazil found a prevalence of low sleep quality of 51.5%, pooling together four studies [39]. In the USA, a survey recruiting a large, multi-university sample of college students found a high rate of poor sleep quality (62%), ranging from 57% among males, to 64% among females [40]. In particular, among American medical students, although the recruited subjects reported sleeping nearly 7 hours per night, the majority of students reported indicators of poor sleep quality. Specifically, attending the first and third year was associated with reporting higher rates of sleep-related issues compared to attending the second and fourth year. First and second year students reported also the highest levels of sleep somnolence. Stratifying according to ethnicity revealed that minority students were more likely to report lower levels of sleep adequacy and sleep quantity and higher levels of sleep somnolence in a statistically significant way when compared with their Caucasian counterparts [41]. An even higher percentage was computed among students in Saudi Arabia, where poor sleep quality was reported by 74.2% of the study participants [42]. Percentages ranging from 58% to 77.78% were reported in other surveys [43,44,45].

The year of the course (clinical versus pre-clinical) was found to be a significant predictor in a study carried out among medical students in Saudi Arabia, where, however, a lower prevalence rate of sleep disturbances was computed (approximately 30%) [46]. Similarly, a study performed in Nigeria found a rate of 32.5% [47], whereas studies conducted in India and Pakistan reported rates varying from 17.3% to 39.5% [48,49]. 

In China, a meta-analysis synthesizing 76 studies involving a sample of 112,939 university students computed an overall pooled prevalence of sleep disturbances of 25.7% (95% CI 22.5–28.9), ranging from 18.1% (95% CI 16.4–20.0) to 24.1% (95% CI 21.0–27.5), depending on the tool utilized. More in detail, the prevalence rates of students dissatisfied with their sleep quality and those suffering from insomnia symptoms were 20.3% (95% CI 13.0–30.3) and 23.6% (95% CI 18.9–29.0), respectively. Subgroup analyses revealed that among the diverse student groups, the category of medical students was particularly prone to sleep-related disorders [50]. This is in line with our findings, showing higher rates among medical and nursing students and lower ones among paramedical students.

This considerable variability in prevalence rates among the studies can be due to the different academic and training conditions and demands, as well as to the students’ personality traits and lifestyles in different countries. This confirms the usefulness of conducting meta-analytical approaches at the country level to better inform local policy- and decision-makers. Furthermore, the study design and the psychometric tool utilized could at least partially account for such contrasting findings.

In any case, poor sleep quality among medical and healthcare professions students represents a global public health issue. Authorities and institutions should make efforts in order to improve students’ quality of life. Students reporting poor sleep are more likely to report anxiety, depression, consumption of drugs, illicit substances, and stimulants [51]. 

Whereas sleep literacy and knowledge appear to be quite satisfactory among medical and allied health professions students, even though some studies report on the contrary scarce knowledge and consistent gaps [8,9], sleep self-awareness is, instead, insufficient, so interventions aimed at increasing self-awareness among students should be considered, in particular for those students at a higher risk. Measures and programs should be targeted and individualized as much as possible [4], and also making use of innovative technologies. A recent systematic review and meta-analysis found that internet-based interventions for university students’ mental health issues, including sleep problems, can have significant small-to-moderate effects [52].

However, our knowledge and our understanding about sleep disturbances among students is scarce and generally comes from studies of low quality, which offer indirect, imprecise, or mixed scientific evidence. As such, high-quality studies are warranted to better capture the determinants of poor sleep quality, given the importance of such a problem and its implications [53].

Concerning the generalizability of our findings to other professions in Iran, such as engineering or architecture students, the comparison is hindered by the dearth of relevant, specific data. Researchers should, as such, explore sleep quality and sleep disturbances among other professional figures, given the importance of such a topic in terms of occupational safety and well-being. A systematic review and meta-analysis among Iranian drivers [54] has computed a prevalence rate of sleep disturbances of 53.4% (95% CI 38.9–67.8), which is well comparable to our estimates.

Our study has some strengths, including its novelty, methodological rigor, and transparency, which enable replication and reproducibility of our findings. Other strengths are given by the lack of publication bias and by the multi-disciplinary nature of our research team.

On the other hand, it suffers from a number of shortcomings, which should be properly acknowledged. The major drawback is given by the high, statistically significant amount of heterogeneity, which is inevitable in meta-analytical studies. Possibly, the methodological differences among the studies and the conditions/settings of the studies could explain, at least partially, such inconsistency. To better study the sources of the heterogeneity, we performed sub-group and meta-regression analyses, although the studies did not provide suitable information for conducting a complete assessment, especially concerning determinants of sleep disturbances. Therefore, we had to limit ourselves to a basic, preliminary qualitative analysis of factors impacting on sleep disturbances. Moreover, there was no possibility of extensively comparing sleep quality between medical and non-medical students, since only a few studies performed such a comparison. Moreover, studies have not been carried out in many provinces of Iran, and this does not provide an overview of the prevalence rate of sleep disturbances among medical and healthcare professions students of the entire country of Iran, but only a partial snapshot of it. Further, the prevalence of sleep disturbances was assessed by means of the overall score of the PSQI, rather than utilizing the scores of the specific components (e.g., sleep latency, sleep duration), that is to say the sub-scores of the PSQI. 

## 5. Conclusions

Poor sleep quality among medical and allied health professions students represents a serious issue and a challenge for educators and policy- and decision-makers, who should make serious efforts to cope with it. Furthermore, based on the aforementioned limitations, future studies investigating sleep disturbances among medical and healthcare professions students should report sufficient data in order to properly examine the major factors affecting sleep quality. 

## Figures and Tables

**Table 1:** The main characteristics of the studies included in the present systematic review and meta-analysis.

First Author	Study Year	City	Mean Age (± Standard Deviation; Minimum–Maximum)	Sample Size	Prevalence Rate (%)
Ghoreishi	2008	Zanjan	23 ± 2.81	224	40.6
Farhadi Nasab	2008	Hamadan	21.73 ± 3.5	150	48.0
Sahraian	2010	Shiraz	21.5 ± 2.67	159	57.2
Eslami Akbar	2011	Jahrom	17–27 *	418	NA
Ghanei	2011	Urmia	21	160	43.1
Mansouri	2012	Tehran	23.4 ± 2.7	277	73.3
Aghajanloo	2012	Zanjan	20.62 ± 1.26	162	86.4
Rezaei Ardani	2012	Mashhad	22.7 ± 2.6	310	39.8
Rambod	2012	Shiraz	NA	87	61.0
Lashkaripour	2012	Zahedan	NA	340	62.4
Moudi	2013	Babol	20–35 *	153	78.8
Ahmadzadeh Vosta	2013	Tehran	26.1 ± 5.1	80	58.8
Alimirzae	2014	Kerman	NA	349	71.0
Mousavi Asl	2015	Yasuj	18–31 *	151	69.95
Foroutan	2015	Shahroud	22.66 ± 0.90	42	64.3
Rezaei	2017	Tehran	22.1 ± 3.6	275	NA
Bahrami	2018	Semnan	21.78 ± 2.91	249	NA

NA: not available; * minimum–maximum.

**Table 2:** Meta-regression analyses based on sample size, year of publication and average age of students. No statistically significant effects could be detected.

Variables	Coefficient	Standard Error	Statistical Significance (*p*-Value)	Lower 95% CI	Upper 95% CI
Sample size	0.0000155	0.0005143	0.976	−0.0010808	0.0011118
Year of publication	−0.0201732	0.0191123	0.308	−0.0609101	0.0205636
Average age of students	0.0375554	0.0314417	0.260	−0.032501	0.1076119

CI: confidence interval.

**Table 3:** Main findings of the sub-group analyses, showing the statistically significant impact on the prevalence rate of sleep disturbances of methodological quality of studies included, sample size, and region where the studies were conducted.

Variables	Number of Studies	Number of Participants	Prevalence Rate and 95%CI	I^2^	Statistical Significance (*p*-Value)
**Methodological quality of the studies included**					
1–8 (weak quality)	3	397	50% (41–60)	73.3%	0.000
9–16 (moderate quality)	5	1219	59% (30–88)	99.44%	0.000
17–22 (good quality)	9	1970	59% (43–75)	98.4%	0.000
**Sample sizes of the studies included**					
Less than 200 participants	9	1144	63% (52–74)	94%	0.000
More than 200 participants	8	2442	52% (31–72)	99.3%	0.000
**Region where the studies included were conducted**					
North	4	785	75% (22–91)	99.2%	0.000
West	6	1006	58% (41–74)	97%	0.000
East	5	1290	52% (32–71)	98.3%	0.000
South	2	505	75% (48–93)	95.9%	0.000

CI: confidence interval; I^2^: heterogeneity.

**Table 4:** A qualitative analysis of determinants of sleep disturbances among medical and health professions students.

Determinants	Number of Studies per Determinant	Number of Studies Reporting a Significant Impact of Determinants on Sleep Disturbances
Poor student dormitories	11 (64.7%)	10 (90.9%)
Age	9 (52.9%)	6 (66.7%)
Family avoidance	9 (52.9%)	8 (88.9%)
Hospital-related activities	8 (47.1%)	8 (100.0%)
Fatigue	7 (41.2%)	4 (57.1%)
High workload and volume of college lessons	7 (41.2%)	5 (71.4%)
Marriage	7 (41.2%)	5 (71.4%)
Depression	6 (35.3%)	3 (50.0%)
Family problems	5 (29.4%)	3 (60.0%)
Sex	5 (29.4%)	3 (60.0%)
Economic problems	4 (23.5%)	2 (50.0%)
Drug use	3 (17.6%)	1 (33.3%)
Anxiety	2 (11.8%)	1 (50.0%)
